# The Sign 4 Little Talkers Intervention to Improve Listening, Understanding, Speaking, and Behavior in Hearing Preschool Children: Outcome Evaluation

**DOI:** 10.2196/15348

**Published:** 2020-06-30

**Authors:** Rosemary Davidson, Gurch Randhawa

**Affiliations:** 1 Institute for Health Research University of Bedfordshire Luton United Kingdom

**Keywords:** sign language, early years, intervention, disadvantage

## Abstract

**Background:**

Gaining age-appropriate proficiency in speech and language in the early years is crucial to later life chances; however, a significant proportion of children fail to meet the expected standards in these early years outcomes when they start school. Factors influencing the development of language and communication include low income, gender, and having English as an additional language (EAL).

**Objective:**

This study aimed to determine whether the Sign 4 Little Talkers (S4LT) program improves key developmental outcomes in hearing preschool children. S4LT was developed to address gaps in the attainment of vocabulary and communication skills in preschool children, identified through routine monitoring of outcomes in early years. Signs were adapted and incorporated into storybooks to improve vocabulary, communication, and behavior in hearing children.

**Methods:**

An evaluation of S4LT was conducted to measure key outcomes pre- and postintervention in 8 early years settings in Luton, United Kingdom. A total of 118 preschool children were tested in 4 early years outcomes domains—listening, speaking, understanding, and managing feelings and behavior—as well as Leuven well-being scales and the number of key words understood and spoken.

**Results:**

Statistically significant results were found for all measures tested: words spoken (*P*<.001) and understood (*P*<.001), speaking (*P*<.001), managing feelings and behavior (*P*<.001), understanding (*P*<.001), listening and attention (*P*<.001), and well-being (*P*<.001). Approximately two-thirds of the children made expected or good progress, often progressing multiple steps in educational attainment after being assessed as developmentally behind at baseline.

**Conclusions:**

The findings reported here suggest that S4LT may help children to catch up with their peers at a crucial stage in development and become *school ready* by improving their command of language and communication as well as learning social skills. Our analysis also highlights specific groups of children who are not responding as well as expected, namely boys with EAL, and who require additional, tailored support.

## Introduction

### Background

As the importance of speech and language ability in later educational outcomes and life chances has been acknowledged, systematic research has tried to evaluate the effectiveness of interventions or programs to improve educational attainment in these areas. A UK Department of Education review of interventions for children who need support with speech, language, and communication found a *sound emerging evidence base* for them [1]. A Cochrane review of the effectiveness of speech and language interventions for children with speech and language delay or disorders described an overall positive effect [2]. Carneiro and Heckman [3] argued that the early development of cognitive and noncognitive skills is key to determining children’s chances of success and that early interventions are much more likely to be beneficial than those targeted at older age groups: “The evidence points to a high return to early interventions and a low return to remedial of compensatory interventions later in the lifecycle” [3].

The UK government has been committed to expanding preschool education in recent years. Currently, all children aged 3 to 4 years in England are entitled to free nursery education or childcare with an approved childcare provider. Children receive 15 hours of free nursery education or childcare from their third birthday, with some parents eligible for 30 hours if employed or getting parental leave (with each parent earning at least the national minimum wage for 16 hours per week). Two-year-old children in England are also entitled to 15 hours of free nursery education with a *Funded 2* childcare place if their parents are in receipt of benefits, if they are looked after by the local council or by guardians, if they have a current statement of special educational needs or are on disability living allowance [4].

### Factors Influencing Development in the Early Years

#### Low Income

Different social environments support language acquisition to varying degrees, depending on the availability of the opportunity for communication to facilitate language acquisition. It has been found that lower-income parents gesture less frequently, with their children starting school with smaller vocabularies than children of a higher socioeconomic status (SES). This is particularly significant as vocabulary is viewed as a key predictor of educational attainment [5]. Children from professional families were found to speak nearly 300 more words per hour when compared with families in receipt of benefits, resulting in a *30-million word gap* [6]. Similarly, children who are surrounded by receptive parents, teachers, and siblings who listen, interact, and respond to facial expressions develop speech at a faster rate [7]. Exposure to high volumes of language enhances learning [8], and current vocabulary makes children more receptive to new learning [9]. Environments rich in cognitive, emotional, and social interactions, where children are exposed to general knowledge, can help children advance quicker [10].

A UK government report highlights the importance of parental behavior [11]. The children of parents who have some form of qualification, who read to their children, and who are interested in their schooling are likely to do better: “…not reading to young children is serving as a proxy for a lack of interest in children’s education, the dominant variable” [11]. The report uses data from the 1970 British birth cohort and concludes that children who perform well academically who are as young as 5 years of age are more likely to escape poverty. Further analysis of the cohort data found that scores at 22 months predicted educational qualifications at 26 years, and this was related to SES. Children of educated or wealthy parents were able to catch up if they had low scores as young children, in contrast with children of lower SES parents who were extremely unlikely to catch up if they had lower scores [12].

Focusing on the shorter term, lower-income children were found to lag behind their higher-income counterparts in vocabulary by 16 months when they start school. The gaps found in language are much larger than the gaps in other cognitive skills [13], and children from the poorest fifth of UK families are nearly 1 year behind their middle-income peers in vocabulary tests at age 5 years when starting school [14]. Children of lower SES and with English as an additional language (EAL) are less skilled in English oral language compared with children of higher SES, English-speaking homes, which then affects academic achievement. The challenges faced by children with EAL are explored further in the following subsection.

#### English as an Additional Language

A study assessing UK primary school children over a 3-year period reported that children learning English had lower levels of vocabulary and comprehension, which is attributed to a lack of fluency when starting school [15]. A later study confirmed these findings, reporting that EAL learners have difficulty understanding written and spoken texts, and have significantly lower levels of vocabulary [16]. Similarly, whereas children with EAL often have good reading skills, limited vocabulary constrains the comprehension of spoken and written texts and therefore support is recommended to develop vocabulary in early years settings [17]. This is in addition to the need to develop appropriate background knowledge in children with EAL to facilitate text comprehension [18]. Comparing samples of bilingual and monolingual speaking children on measures of vocabulary and grammar, monolingual children were significantly more advanced in vocabulary and grammar, but comparable in terms of total vocabulary size [19].

#### Gender

In the late 1980s, a meta-analysis of gender differences in verbal ability reported that differences no longer exist, with females scoring only slightly better than males [20]. However, later work showed that symbolic gestures develop alongside children’s early words, that girls tended to rely more heavily on such gestures than boys, and that structured parent-child interaction is important in developing these gestures and is positively related to verbal vocabulary development [21]. Nevertheless, concern has increased over girls outperforming boys educationally. However, the evidence appears to be more nuanced for school-aged children. For example, a large-scale longitudinal study found that girls and boys outperform each other depending on the learning domains investigated. Similarly, an international study investigating sex differences in the Program for International Student Assessment achievement and national measures of gender equality found inconsistencies across assessments. However, in terms of overall achievement across reading, mathematics, and science literacy, girls outperformed boys in 70% of the countries under study. Gender differences become most pronounced in higher education, where male participation has dropped substantially as female participation has increased. This has been reported both in the United Kingdom and worldwide [22].

#### Emotional Literacy

Socioemotional development is increasingly acknowledged as important for future life opportunities. *Effective mastery* of social and emotional skills supports the attainment of key life outcomes such as *good health and social well-being, educational attainment and employment, and the avoidance of behavioral and social difficulties* [23]. This is in the context of increasing concern over children’s mental health and well-being [24]. Gesturing has been proposed as a *therapeutic communication tool* to help children express emotions and construct an understanding of their own internal states [25].

Goodman et al [26] linked social, emotional, and cognitive skills recorded at 10 years of age from the British Cohort Study of those born in 1970 with experiences at 42 years of age. Therefore, developing a good range of cognitive, social, and emotional skills in childhood was viewed as important for success in adult life, including encouraging good emotional well-being, self-regulation, and a sense of self-efficacy. Moreover, psychological problems experienced in childhood affect the ability to work in adulthood, earning power, marital stability, and intergenerational and within-generation social mobility [27]. In terms of ensuring school readiness, it is argued that developing preschool children’s socioemotional competence and language skills help them to adjust to junior school. This is particularly important for at-risk children as a way of ensuring school readiness [28].

### Evidence of the Effectiveness of Sign Language or Gesturing in Children

Recent guidance from the Education Endowment Foundation recommends the prioritization of the development of communication and language and embedding opportunities to develop self-regulation [29]. The use of sign language to encourage speech and vocabulary range in hearing children has been investigated in recent years, suggesting that gesturing is a precursor to speech [30], and in the relationship between motor skills and language development [31]. A multisensory learning approach using visual aids, hearing, speaking, and signing helped preschool children retain more words and phonetic sounds [32]. Cook and Goldin-Meadow [33] found that gesturing during teaching and encouraging children to mirror them increased engagement and interaction with learning. Daniels [34] found that young hearing children significantly increased their vocabularies when teachers used sign language when compared with conventionally taught children and that such positive effects were maintained throughout the following school year [34]. Similar significant gains in vocabulary were reported when hearing children were taught to incorporate American Sign Language [35]. Elsewhere, gesturing at 18 months was found to predict vocabulary at 42 months, and gesture and speech combinations at 18 months predicted the degree of sentence complexity at 42 months [36]. Brain scanning research has found that symbolic gesturing, signs, and words activate the same brain areas, suggesting that word learning is enhanced with activity such as gesturing conveying meaning, facilitating word learning [37,38].

### The Sign 4 Little Talkers Intervention

Sign 4 Little Talkers (S4LT) was developed to address gaps in the attainment of vocabulary and communication skills in preschool children, which were identified through routine monitoring of early years outcomes (EYOs) by Luton Borough Council, United Kingdom. The town faces additional challenges due to higher than average levels of deprivation, a transient population, and multiple languages being spoken. The S4LT intervention consists of 5 books, *Feelings*, *The Lost Teddy*, *I want that!*, *Can I go to the park?*, and *It is too noisy* [39], which depict 2 characters, Zak and Zoe. Zak and Zoe are also dolls that are used during story sessions to engage children. A DVD and poster are also available for early years settings to train practitioners to use S4LT stories and signing.

Signs are adapted from British Sign Language to increase vocabulary in hearing children. The S4LT books are designed to improve communication in preschool children, express their emotions, and start regulating their own behavior. Stories depict different situations, and the emotions that children might feel, accompanied by signs, for example, “Zoe is excited. Why is she excited? Because she‘s on the roundabout!” By reading the stories, parents and carers are engaged in promoting positive behavior such as sharing, turn taking, using linking words to form longer and more complex sentences as well as those needed in social interactions such as *please* and *thank you*. Parents and carers are encouraged to talk to children about how useful it is to use hand gestures when telling a story to help them remember vocabulary and to encourage children to say and sign words with them.

We investigated the effectiveness of the S4LT intervention for preschool children [40]. S4LT is one of many Sign 4 programs developed to improve various aspects of child development. An evaluation of another of these, Sign 4 Big Feelings, designed to support children with challenging behavior was also conducted [41]. This is part of a wider evaluation of services for children aged under 5 years in Luton, United Kingdom [42-45]. Underlying the S4LT intervention is the hypothesis that the adoption of sign language by hearing children accelerates proficiency in speech, language acquisition, and well-being, thereby improving outcomes in these developmental areas at a better-than-expected rate. An analysis of pre- and post-S4LT outcome data collected from Luton early years settings aims to answer the following research question: Does the S4LT intervention improve language, communication, and well-being outcomes in preschool children?

## Methods

### Ethics Approval and Consent to Participate

This study was approved by the University of Bedfordshire Research Ethics Committee (UREC104) on April 10, 2017. Written consent was obtained from the parents.

### Availability of Data and Materials

The data sets used and analyzed during this study are available from the corresponding author upon reasonable request.

### Implementation

The S4LT intervention was introduced into 8 early years settings (nursery schools, preschools, or kindergartens) in Luton. These settings were chosen because they reported lower than expected progress in the attainment of communication and language skills as monitored by the Early Years Foundation stage outcome data (EYOs) routinely collected and inputted into Luton Borough Council’s tracking system. This targeted strategy was adopted to ensure that children with the greatest level of need could benefit from the intervention. Children attending these early years settings do not do well when compared with both the Luton and UK national average in communication, language, and managing feelings and behavior as measured by the Early Years Foundation stage profile. This statutory framework sets standards for the development and care of children aged below 5 years in the United Kingdom to ensure effective learning and development and ensure that they are ready to start school.

Principals or managers in the 8 settings signed a memorandum of understanding, setting out expectations regarding the implementation of S4LT. A training session was organized at each of the 8 settings for staff to familiarize themselves with the books, learn the signs, and practice with each other. The intervention was to be incorporated into daily routines such as story times, giving children the opportunity to learn the Sign 4 stories and sign language gradually on a daily basis. Staff in each early years setting were asked to identify 10 children who were assessed as below expected levels of development and therefore judged to be most in need of targeted help to catch up with their peers. The chosen children received additional support with extra story sessions, and their parents were invited to an S4LT session with their children to learn the stories and signs. They were given a set of 2 S4LT books and dolls to use with their children at home. Data were collected from 10 children per setting before the introduction of S4LT and again after 2 terms, starting autumn 2016 and ending spring 2017 (approximately 6 months).

### Outcome Measures

#### Early Years Outcome Developmental Bandings

Nursery staff in Luton use EYO developmental bands to monitor children on a termly basis. Children are placed in age bands according to their level of development (ie, 22-36 months, 30-50 months). Children are placed in a specific age band, and each band is subdivided into c=low, b=secure, and a=high, until they reach the early learning goal (ELG), which is the expected level of learning and development for children at the end of the reception year at school (Table 1). Assessment is made in a number of different areas of learning, but for the purposes of this study, we were interested in 4 particular domains: listening, understanding, speaking, and managing feelings and behavior. For example, a child may be rated in the 22- to 36-month band at *high*, indicating they are in the upper end of educational attainment in that particular age band. The banding may not reflect chronological age as it depends on individual progress. Therefore, a child aged 28 months may be put into the 30- to 50-month banding if they are above typical levels of development for their age and conversely in a lower band than their age if they are below typical levels. Children are expected to progress to 1 developmental stage per term, for example, 30-50c (low) to 30-50b (secure). As data collection pre- and postintervention was over 2 terms, children would be expected to progress 2 bands on average.

**Table 1:** Early years outcomes up to early learning goals.

Developmental stage	EYOs^a^ to ELGs^b^
1	8-20c
2	8-20b
3	8-20a
4	16-26c
5	16-26b
6	16-26a
7	22-36c
8	22-36b
9	22-36a
10	30-50c
11	30-50b
12	30-50a
13	40-60c
14	40-60b
15	40-60a
16	ELG1
17	ELG2
18	ELG3

^a^EYOs: early years outcomes.

^b^ELGs: early learning goals.

#### Leuven Well-Being Scales

To explore hypothesized links between low levels of well-being, involvement, and compromised development [46,47], well-being was measured. To ascertain if children’s well-being improved after the intervention period, the Leuven well-being scale was employed [48] and is used by early years professionals in Luton.

#### Number of Words Understood and Spoken

The number of words understood and spoken from 42 keywords featured in the S4LT story books were recorded pre- and postintervention (happy, sad, angry, frustrated, disappointed, frightened, worried, excited, upset, tired, hungry, sorry, please, thank you, calm down, sit down, well done, gentle, wait, stop, share, kind, your turn, listen, why, because, first, next, finally, so, what, who, quiet, loud, where, hiding, dangerous, safe, crying, secret, shouting, proud). The demographic information collected included gender, child’s age in months, EAL, and if the child had a funded nursery place (Funded 2), which was also used as an indicator of deprivation.

## Results

### Summary of the Data Set: Participants

Data from 119 children were collected (Table 2), with more boys (65/119, 54.6%) than girls (44/119, 37.0%). Just under 48% of children were in the 2- to 3-year age band and 41.2% (49/119) were in the 3- to 4-year age band. Some of the data were marked *unknown* where forms were incomplete but contained sufficient data to be included. Some measures had totals of less than 119 children where data were missing. Just over one-third of the children had EAL, and over 70.0% had a funded early years childcare place (Funded 2).

A total of 118 EYO assessments in 4 domains were completed (listening, understanding, speaking, and managing feelings and behavior); 108 assessments of keywords understood and spoken pre- and postintervention, and 46 Leuven well-being scales pre- and postintervention (Table 3) These measures are described under the previous outcomes section. Baseline data were collected in September 2016, and follow-up data were collected 6 months later in March 2017. Descriptive statistics, a paired samples two-tailed *t* test, and correlations were completed.

**Table 2:** Preschool children by age, gender, English as an additional language, and Funded 2 status.

Children		Values, n (%)
**Gender**		
	Male	65 (54.6)
	Female	44 (37.0)
	Unknown	10 (8.4)
**EAL^a^**		
	Yes	40 (33.6)
	No	78 (65.5)
	Unknown	1 (0.8)
**Age (years)**		
	1-2	3 (2.5)
	2-3	57 (47.9)
	3-4	49 (41.2)
	Unknown	10 (8.4)
**Funded 2**		
	Yes	72 (60.5)
	No	37 (31.1)
	Unknown	10 (8.4)

^a^EAL: English as an additional language.

**Table 3:** Summary of the Sign 4 Little Talkers data set.

Variables	n	EYO^a^ progress (range)	Value, mean (SD)
Listening_Progress_Made	118	−2.00 to 6.00	2.3051 (1.51634)
Understanding_Prog_Made	118	−1.00 to 7.00	2.4661 (1.58347)
Speaking_Progress_Made	118	0 to6.00	2.4492 (1.50553)
Feelings_Progress_Made	118	−2.00 to 6.00	2.5508 (1.54476)
Words Autumn1_Understanding	108	0 to 16.00	6.3796 (4.04118)
Words Autumn1_Speaking	108	0 to 16.00	4.1852 (3.92725)
Words Spring2_Understanding	108	3.00 to 16.00	11.3704 (3.58760)
Words Spring2_Speaking	108	0 to 16.00	9.6296 (4.47964)
Leuven wellbeing_Autumn1	46	1.00 to 5.00	2.8913 (1.07968)
Leuven wellbeing_Spring2	46	2.00 to 5.00	4.0217 (.71458)
EYO Aut1_Listening	118	0 to 14.00	7.7203 (2.94939)
EYO Aut1_Understanding	118	0 to 14.00	7.2797 (2.97536)
EYO Aut1_Speaking	118	0 to 14.00	6.5678 (2.87779)
EYO Aut1_Feeling	118	0 to 13.00	6.9153 (2.78757)
EYO Spr2_Listening	118	0 to 15.00	10.0254 (2.80860)
EYO Spr2_Understanding	118	0 to 15.00	9.7373 (2.88061)
EYO Spr2_Speaking	118	0 to 15.00	9.0169 (2.94387)
EYO Spr2_Feeling	118	0 to 14.00	9.4661 (2.90794)

^a^EYO: early years outcome.

### Paired Sample t Test

A paired sample *t* test was conducted to ascertain any statistically significant difference in mean scores before and after the introduction of the S4LT intervention. Statistically significant differences in mean scores were found for each of the 7 pre- and postpairs tested (Table 4). The mean of keywords understood in the spring term (mean 11.37, SD 3.59) was significantly higher than that of the autumn term (mean 6.38, SD 4.04; t_107_=16.44; *P*<.001; Cohen *d*=1.58). The mean of keywords spoken was significantly higher in the spring term (mean 9.63, SD 4.48) than that of the previous autumn term (mean 4.18, SD 3.93; t_107_=15.21; *P*<.001; Cohen *d*=1.47). The mean Leuven well-being scale in the spring term (mean 4.02, SD 0.71) was significantly higher than that of the autumn term (mean 2.89, SD 1.08; t_45_=10.24; *P*<.001; Cohen *d*=1.53). The mean of EYO listening and attention were significantly higher in the spring term (mean 10.02, SD 2.81) than that of the autumn term (mean 7.72, SD 2.95; t_117_=16.51; *P*<.001; Cohen *d*=1.52). The mean of EYO understanding in the spring term (mean 9.74, SD 2.88) was significantly higher than that of the autumn term (mean 7.28, SD 2.97; t_117_=16.92; *P*<.001, Cohen *d*=1.50). The mean of EYO speaking in the spring term (mean 9.02, SD 2.94) was significantly higher than that of the autumn term (mean 6.57, SD 2.88; t_117_=17.67; *P*<.001; Cohen *d*=1.62). The mean of EYO managing feelings and behavior was significantly higher in the spring term (mean 9.47, SD 2.91) than that of the autumn term (mean 6.91, SD 2.79; t_117_=17.94; *P*<.001; Cohen *d*=1.65). All 7 tested pairs had large effect sizes, indicating large statistically significant differences.

**Table 4:** Paired sample t test: words understood and spoken, well-being, and early years outcomes.

Number and pairs		Paired differences			*t* test (*df*)		Significance (two-tailed)	
		Mean (SD)	SE	95% CI				
Pair 1	Words Aut1 Understand—Words Spr2 Under	−4.99 (3.15)	0.30	−5.59 to −4.39		−16.44 (107)		0
Pair 2	Words Aut1 Say—Words Spr2 Say	−5.44 (3.72)	0.36	−6.15 to −4.73		−15.21 (107)		0
Pair 3	Leuven Wellbeing Aut1—Leuven Well Spr2	−1.13 (.75)	0.11	−1.35 to −0.91		−10.24 (45)		0
Pair 4	EYO^a^ Aut1 Listening—EYO Spr2 Listening	−2.31 (1.52)	0.14	−2.58 to −2.03		−16.51 (117)		0
Pair 5	EYO Aut1 Understanding—EYO Spr2 Under	−2.46 (1.58)	0.15	−2.75 to −2.17		−16.92 (117)		0
Pair 6	EYO Aut1 Speaking—EYO Spr2 Speaking	−2.45 (1.51)	0.14	−2.72 to −2.17		−17.67 (117)		0
Pair 7	EYO Aut1 Feelings—EYO Spr2 Feelings	−2.55 (1.55)	0.14	−2.83 to −2.27		−17.94 (117)		0

^a^EYO: early years outcome.

### Correlations

A correlation analysis was run with children’s age, the 4 EYO domains, Leuven well-being scales, and words understood and spoken (Table 5). There was a positive, statistically significant relationship between age and the 4 EYO domains: listening and attention (Pearson correlation coefficient *r*_109_=0.49; *P*<.001); understanding (*r*_109_=0.52; *P*<.001); speaking (*r*_109_=0.48; *P*<.001); managing feelings and behavior (*r*_109_=0.59; *P*<.001); and words spoken (*r*_108_=0.20; *P*<.03). No relationship was found between age and the Leuven well-being scales and words understood.

A positive, statistically significant relationship was found between each of the EYO domains. For example, listening and understanding (*r*_118_=0.94; *P*<.001) and between speaking and managing feelings and behavior (*r*_118_=0.87; *P*<.001). A positive, statistically significant relationship was also found between the Leuven well-being scale and words understood (*r*_46_=0.39; *P*<.007) and spoken (*r*_46_=0.39; *P*<.008).

**Table 5:** Pearson correlations: words understood and spoken, well-being, and early years outcomes.

Variables		Age	EYO^a^ Spr2 List&Att	EYO Spr2 Under	EYO Spr2 Speak	EYO Spr2 Feel	Leuven Spr2	Words Spr2 Under	Words Spr2 Say
	Pearson correlation, *r*	1.00	0.49	0.52	0.48	0.59	−0.08	0.16	0.20
	Significance (two-tailed)	N/A^b^	0.00	0.00	0.00	0.00	0.60	0.09	0.03
	Number of participants, n	109	109	109	109	109	46	108	108
**EYO spring (second term) listening and attention**									
	Pearson correlation, *r*	N/A	1.00	0.94	0.89	0.91	−0.26	0.03	0.07
	Significance (two-tailed)	N/A	N/A	0.00	0.00	0.00	0.08	0.78	0.45
	Number of participants, N	N/A	118	118	118	118	46	108	108
**EYO spring (second term) understanding**									
	Pearson correlation, *r*	N/A	N/A	1.00	0.92	0.92	−0.24	0.04	0.05
	Significance (two-tailed)	N/A	N/A	N/A	0.00	0.00	0.11	0.66	0.60
	Number of participants, N	N/A	N/A	118	118	118	46	108	108
**EYO spring (second term) speaking**									
	Pearson correlation, *r*	N/A	N/A	N/A	1.00	0.87	−0.27	0.04	0.05
	Significance (two-tailed)	N/A	N/A	N/A	N/A	0.00	0.07	0.66	0.58
	Number of participants, N	N/A	N/A	N/A	118	118	46	108	108
**EYO spring (second term) feelings and behavior**									
	Pearson correlation, *r*	N/A	N/A	N/A	N/A	1.00	−0.24	0.09	0.11
	Significance (two-tailed)	N/A	N/A	N/A	N/A	N/A	0.11	0.37	0.26
	Number of participants, N	N/A	N/A	N/A	N/A	118	46	108	108
**Leuven well-being spring (second term)**									
	Pearson correlation, *r*	N/A	N/A	N/A	N/A	N/A	1.00	0.39	0.39
	Significance (two-tailed)	N/A	N/A	N/A	N/A	N/A	N/A	0.01	0.01
	Number of participants, n	N/A	N/A	N/A	N/A	N/A	46	46	46
**Words spring (second term) understood**									
	Pearson correlation, *r*	N/A	N/A	N/A	N/A	N/A	N/A	1.00	0.66
	Significance (two-tailed)	N/A	N/A	N/A	N/A	N/A	N/A	N/A	0.00
	Number of participants, n	N/A	N/A	N/A	N/A	N/A	N/A	108	108
**Words spring (second term) spoken**									
	Pearson correlation, *r*	N/A	N/A	N/A	N/A	N/A	N/A	N/A	1.00
	Significance (two-tailed)	N/A	N/A	N/A	N/A	N/A	N/A	N/A	N/A
	Number of participants, n	N/A	N/A	N/A	N/A	N/A	N/A	N/A	108

^a^EYO: early years outcome.

^b^N/A: not applicable.

### Progress of Early Years Outcomes by Stage

Control data collected from the same academic year showed the average progress of children in Luton over 2 terms, with 1 or 2 steps made in each domain under study (Table 6). Children are typically expected to progress 1 step per term and therefore fell short of the expected progress in listening and attention and speaking over the 2 terms reported here. Table 7 shows the progress made by children in the study sample in each EYO domain. Approximately two-third of children made expected (2 stages over 2 terms) or good progress in listening (67%), understanding (71%), and speaking (69.5%). Nearly four-fifth (79%) made expected or good progress in managing feelings and behavior.

In terms of listening, children with EAL made less progress than English speakers; boys made less progress than girls (Table 8). Boys with a Funded 2 childcare place made significantly more progress than nonfunded boys; nonfunded girls made slightly more progress than those with funded places (Table 9). With understanding, boys with EAL made the least progress. Girls made more progress than boys overall; children with EAL made less progress than their peers did (Table 8). Children in Funded 2 childcare places made better-than-expected progress (more than 2 levels) and outperformed nonfunded children in terms of understanding; overall, boys made less progress (Table 9).

In terms of managing feelings and behavior, boys made less progress than girls, with children with EAL doing less well overall (Table 8). However, boys in Funded 2 childcare places progressed more than their nonfunded peers, whereas nonfunded girls progressed slightly more than funded girls (Table 9). In the final EYO area, speaking, girls progressed more than boys, with children with EAL behind their English-speaking peers (Table 8). Children in Funded 2 childcare places made more progress in speaking than nonfunded children (Table 9). The Leuven well-being scales pre- and postintervention show a shift up the scale. The majority of children were assessed as moderate in the autumn term, shifting to *high* in the spring term, with girls proportionately moving further up the scale than boys (Table 10).

**Table 6:** Control data: average steps progress of children over 2 school terms.

EYO^a^ domain	Autumn term 1				Spring term 2				Steps progress
	Total number of pupils, N	Below expected progress, n	At expected progress, n	Above expected progress, n	Total number of pupils, N	Below, n	At, n	Above, n	
Listening and attention	413	34	45	20	498	26	38	36	1
Understanding	413	41	41	18	498	36	34	30	2
Speaking	404	47	40	14	498	43	30	26	1
Managing feelings and behavior	394	40	48	12	498	38	38	25	2

^a^EYO: early years outcome.

**Table 7:** Progress made by children in each early years outcome domain.

EYO^a^ domain	EYO stages progressed									
	2− stages	1− stage	0 stages	1+ stage	2+ stages (expected progress)	3+ stages	4+ stages	5+ stages	6+ stages	7+ stages
Listening	2	N/A^b^	11	28	25	32	12	4	3	N/A
Understanding	N/A	1	12	20	23	34	16	5	3	2
Managing feelings and behavior	1	1	7	15	37	28	13	11	4	N/A
Speaking	N/A	N/A	10	26	23	35	11	7	5	N/A

^a^EYO: early years outcome.

^b^N/A: not applicable.

**Table 8:** Progress made by children with and without English as an additional language in each early years outcome domain.

EYO^a^ domain	English as an additional language				
	Male			Female	
	Yes	No	Yes		No
Listening	1.5	2.5	2.0		3.0
Understanding	1.5	2.8	2.5		2.9
Managing feelings and behavior	1.8	2.5	2.4		3.0
Speaking	1.7	2.5	2.2		2.8

^a^EYO: early years outcome.

**Table 9:** Progress made by children with and without a Funded 2 childcare place in each early years outcome domain.

EYO^a^ domain	Funded 2 place				
	Male			Female	
	Yes	No	Yes		No
Listening	2.5	1.6	2.5		2.8
Understanding	2.5	2.0	2.7		2.6
Managing feelings	2.4	1.9	2.7		3.0
Speaking	2.5	2.0	2.8		2.2

^a^EYO: early years outcome.

**Table 10:** Well-being of children in autumn and spring terms as assessed by the Leuven scale.

Leuven term		Scale					Values, mean (SD)
		Extremely low	Low	Moderate	High	Extremely high	
**Autumn**							
	Male	N/A^a^	5	14	3	3	3.16 (0.89)
	Female	5	5	6	4	1	2.57 (1.20)
**Spring**							
	Male	N/A	N/A	4	15	6	4.12 (0.66)
	Female	N/A	1	4	12	4	3.90 (0.76)

^a^N/A: not applicable.

## Discussion

### Principal Findings

Statistically significant differences in mean scores were found in each of the pairs tested pre- and postintervention: words understood and spoken, Leuven well-being scales, and the 4 EYO domains. The majority of children made expected progress or better in terms of EYO stages (67% listening, 71% understanding, 69.5% speaking, and 79% managing feelings and behavior), with many progressing multiple steps. The mean progress in each domain was between 2.3 and 2.5 steps (Table 3) and therefore better than that reported in the control data (Table 6), particularly in relation to listening and attention and speaking, where an average of 1 step progress was made. Children monitored as part of the intervention were chosen because they were identified as making less-than-expected progress, and it could be argued that they were even further behind as they were identified as most in need of help by early years professionals working with them on a daily basis. This makes the multiple *steps* of progress made by the majority of children in the S4LT intervention even more notable.

The correlation analysis found a positive, statistically significant relationship between age and the 4 EYO domains, suggesting that as age increases, so does the degree of educational attainment. A statistically significant relationship was also found between each of the 4 EYOs, suggesting that progression in one area is related to progression in the others, which, in terms of understanding, listening, managing feelings and behavior, and speaking, would make sense given the interdependence between them as children gain core skills. This finding was confirmed by early years professionals who routinely observe and therefore would expect children to make progress across all domains after an initial advance in one developmental area as they are inextricably linked. The positive, statistically significant relationship between well-being and words understood and spoken suggests that the ability to communicate and be understood enhances well-being in the children under study. Well-being as measured by the Leuven scale appeared to improve markedly, although caution is advised due to the smaller subgroup of children who were measured.

Although the majority of children made better-than-expected progress, of note is a sizeable minority who made less-than-expected progress or who regressed (33% listening, 30.5% speaking, 29% understanding, and 21% managing feelings and behavior). Possible explanations put forward by early years staff based at the study sites suggest that this may be because of undiagnosed learning difficulty, health issues such as hearing problems, or illness resulting in frequent absences, severe behavioral problems, difficulties at home, and/or an unstable home environment.

### Limitations

A relatively small sample size was collected overall, with the Leuven well-being scale data being particularly limited. This was because not all early years practitioners in Luton were trained to use this particular scale to assess well-being. At the 2-term duration, the intervention period was viewed as quite brief, and this was remarked on by some early years professionals. Timings were decided by the Sign 4 team and the University of Bedfordshire as part of an agreed timeline to report on results in a defined period. Future work would benefit from longer time frames and a longitudinal approach, which would be possible given the type of data collected by Luton Borough Council over time.

In terms of steps taken during EYO progression, caution is advised for children who made multiple steps of progress (smaller numbers moved up to 5-7 stages). However, practitioners working in these settings view it as possible for children to change quite drastically with the right help, support, and encouragement. A forthcoming process study of S4LT, with an analysis of interviews with parents, staff, and stakeholders, will examine lay accounts of the intervention and perceptions of progress made. Regarding fidelity of delivery, the same signing trainer visited all the settings, working with staff, parents, and children. The degree to which settings adopted S4LT and the motivation of the staff to sign would most likely vary. However, inspection of the data did not show much variability between the settings. The assessment of children in terms of EYOs is subject to monitoring by Luton Borough Council. Staff are trained regularly and are moderated to reduce inconsistencies in assessment as much as possible.

This study is concerned with progress measured in terms of steps of EYO developmental bands rather than final results; therefore, these children may still be behind their peers developmentally despite making considerable progress. Nevertheless, the children were selected because they were below the expected levels of development, and within this context, the progress made by the majority is notable. An exception was boys with EAL who made the least progress, suggesting that they require additional, intensive, and tailored support to catch up with their peers and reduce inequalities in educational attainment as much as possible.

### Previous Research and Theory

Children with EAL have been found to have lower levels of vocabulary and comprehension [15,16], potentially putting them at a disadvantage both in the short and long term. The data presented here show that children with EAL made less progress than their English-speaking peers, and boys with EAL made less progress than girls with EAL. Significantly, girls with EAL are making expected progress (2 stages) in each domain after the intervention, whereas boys with EAL are yet to reach this milestone. Nevertheless, they may have made considerable progress given their ability at a given time, and they may catch up more over a longer intervention period.

Although gender differences in educational attainment become apparent as children progress through secondary school and are most pronounced in the university years [22], monitoring of EYOs shows that girls continue to perform better than boys in all early years key areas (76.5% of girls reached the expected levels in all ELGs versus 61.8% of boys) [49]. Our data show that girls outperform boys to varying degrees depending on the EYO domain; however, certain groups of boys are making expected or good progress in some areas. Of note are the boys in Funded 2 childcare places, who outperformed nonfunded boys in listening, understanding, and managing feelings and behavior, and were close to the progress made overall by the girls.

We know that children from lower-income families are at a disadvantage in terms of vocabulary and that the command of vocabulary is a key predictor of educational attainment [5]. The word gap, when compared with professional parents, is particularly stark [6]. Of course, factors such as EAL, gender, and deprivation are not mutually exclusive and children in this study will fall into multiple categories, such as having EAL and low income; therefore, potential risk factors may intersect and further hamper development.

Recent UK data show that over a quarter (28%) of 4- and 5-year-old children lack early communication skills [49]. Arguably, the children participating in the evaluation of S4LT reflect the 28% who are making less-than-expected progress and who may be given the opportunity to make significant advances toward expected progress when such targeted support is given. We acknowledge that there is emerging evidence showing the benefit of interventions for children in need of support with speech, language, and communication [1]. Recent guidance on preparing children for literacy recommends prioritizing the development of communication and language, emphasizing the vital role that adults play in helping children to extend their vocabulary as well as instilling self-regulation [29].

### Conclusions

The findings from this evaluation suggest that S4LT is a tangible, effective approach to help children to catch up with their peers at a crucial stage in development and help them to become school ready by improving their command of language and communication as well as learning social skills. Our analysis also highlights specific groups of children who are not responding as well as expected, namely boys with EAL, and who require additional, tailored support.

## Abbreviations

EAL: English as an additional language
ELG: early learning goal
EYO: early years outcome
S4LT: Sign 4 Little Talkers
SES: socioeconomic status
